# Depressive symptoms, avoidant coping, and alcohol use: differences based on gender and posttraumatic stress disorder in emerging adults

**DOI:** 10.1007/s12144-024-06150-x

**Published:** 2024-06-01

**Authors:** Carla Kmett Danielson, Austin M. Hahn, Kaitlin E. Bountress, Amanda K. Gilmore, Lydia Roos, Zachary W. Adams, Charli M. Kirby, Ananda B. Amstadter

**Affiliations:** 1 Department of Psychiatry & Behavioral Science, Medical University of South Carolina, Charleston, SC 29425, USA; 2 Virginia Institute for Psychiatry and Behavioral Genetics, Richmond, VA, USA; 3 Department of Health Policy & Behavioral Sciences, School of Public Health, Georgia State University, Atlanta, GA, USA; 4 Department of Psychiatry and Biobehavioral Sciences, University of California, Los Angeles, CA, USA; 5 EvolveWell Research Partners, Cincinnati, OH, USA; 6 Department of Psychiatry, Indiana University School of Medicine, Indianapolis, IN, USA

**Keywords:** PTSD, Depressive symptoms, Avoidance, Alcohol use disorder, Trauma, Coping, Emerging adulthood

## Abstract

Trauma exposure and alcohol use often co-occur. Unveiling predictors of drinking behavior, including among those with varying levels of trauma exposure, can inform behavioral health prevention and treatment efforts in at-risk populations. The current study examined associations between depressive symptoms, avoidant coping, gender, and alcohol use among emerging adults with and without trauma exposure and posttraumatic stress disorder (PTSD). Participants were 238 emerging adults between the ages of 21 and 30 years (*M* = 24.75; *SD* = 2.61) in one of three groups: trauma-exposed with PTSD (*n* = 70); trauma-exposed with no PTSD (*n* = 83); or a no trauma (control) group (*n* = 85). Demographics, parental alcohol problems, depressive symptoms, and avoidant coping were examined as predictors of drinks per drinking day. Chi-square, t-test, bivariate, and group path analysis were conducted. Among participants, men consumed greater amounts of alcohol than women across all three groups. Group assignment based on trauma history and PTSD significantly moderated the association between avoidant coping and alcohol use such that avoidant coping had a significant effect on alcohol use among participants in the trauma-exposed and PTSD groups. There was also a significant group × gender × avoidant coping interaction such that, among participants in the control group, men had attenuated alcohol use at low levels of avoidant coping and increased at high levels of avoidant coping. No effects of race were observed. Results highlight the importance of avoidant coping as a risk factor for problematic drinking, unveiling a specific intervention target for reducing co-occurring PTSD and problematic alcohol use.

Alcohol use is common among adults, with approximately two-thirds of adults drinking alcohol in the past year and approximately one-fourth of adults engaging in binge drinking, defined as five or more drinks on the same occasion in the past 30 days (Substance Abuse and Mental Health Service Administration, 2023a, b). Alcohol-related consequences for emerging adults are particularly problematic, including legal, financial, emotional, and medical consequences. Posttraumatic stress disorder (PTSD) and alcohol use often co-occur, and co-occurring PTSD and substance use is associated with greater functional impairment and higher rates of other mental health disorders compared to those with PTSD only (Brady et al., 2004; Riggs, 2003). It is essential to understand the factors underlying this association in order to design and evaluate targeted intervention programs aimed at reducing the burden of alcohol use and traumatic stress. One theoretical model for explaining this association, the self-medication hypothesis, posits that alcohol use and PTSD frequently co-occur because many individuals use alcohol to help mitigate negative emotional experiences associated with mental health disorders (for review, see Hawn et al., 2020). Daily diary studies have supported this hypothesis in that adults with PTSD are more likely to drink on days when they experience more symptoms or more negative affect (Dvorak et al., 2014; Kaysen et al., 2014; Simpson et al., 2014). Further, a tendency to drink alcohol to cope with distress appears to mediate the association between PTSD symptoms and alcohol use in trauma-exposed adults (e.g., Kaysen et al., 2007; Yeater et al., 2010).

Consistent with the self-medication hypothesis, many young people who experience trauma have subsequent increased alcohol use (Danielson et al., 2010; Stappenbeck et al., 2013; Wilsnack et al., 2004). Yet increases in drinking following a traumatic event are not consistently observed (Messman-Moore et al., 2009; Mouilso et al., 2012; Ullman & Najdowski, 2009; Walsh et al., 2012). Therefore, elucidating differential factors that are associated with more alcohol use subsequent to a traumatic event exposure and or development of PTSD is essential. Individual differences, use of avoidant coping strategies, and mental health symptoms all may contribute to the inconsistencies in the prior literature in the association between trauma and subsequent alcohol use.

## Individual differences

Gender and race may be related to differential associations between trauma and alcohol use. Examining these two important individual variables could help explain why some trauma-exposed young people engage in problematic use of alcohol (e.g., to cope with symptoms associated with PTSD), while others do not. There are consistent differences in alcohol use and PTSD based on gender and race. For example, research focusing on gender differences suggests that men report higher rates of drinking than women (Erol & Karpyak, 2015), whereas women are more likely to have PTSD compared to men (Tolin & Foa, 2008). With regard to race, some studies have shown that some African Americans may engage in alcohol use less than European Americans, yet tend to have more alcohol-related negative consequences (Gelernter et al., 2014; Zapolski et al., 2014). In regard to PTSD, a nationally representative sample found that African Americans are more likely to have PTSD in their lifetime compared to European Americans (Alegría et al., 2013). It is important to note that while some research may suggest there may be differences related to gender and race, the authors are not suggesting that gender and racial identities *cause* PTSD. Rather, individuals from minoritized backgrounds are often targeted for violence and experience disproportionate levels of stress due to discrimination, which in turn may result in higher levels of PTSD.

Additionally, there is a wealth of evidence that alcohol use disorder (AUD) is highly familial. Genetically informative epidemiologic designs suggest that the cross-generational transmission of risk of AUD from parents to offspring is from both genetic and environmental (i.e., rearing) effects (Kendler et al., 2015). Thus, examination of familial history of AUD is needed.

## Avoidant coping and mental health symptoms

Avoidance is a common behavior after experiencing a traumatic event and can lead to or denote the development of PTSD. A high level of avoidant coping also is associated with alcohol use disorders among individuals with PTSD (Grosso et al., 2014). However, this association differs by gender. Men and women report equal drinking rates if they are low on avoidant coping, but men who rely on avoidant coping are more likely to drink than women who rely on avoidant coping (Cooper et al., 1992). Further, some studies have suggested that racial differences may exist in the association between avoidant coping and alcohol. For example, the association between the use of coping strategies and alcohol use was found to differ by race (Aldridge-Gerry et al., 2011), where minimization of stressors was associated with more alcohol use in Black Americans, use of religious coping was associated with less alcohol use in Black Americans, and use of problem-focused coping was associated with less alcohol use in White Americans. These differences related to race are likely a reflection of differential experiences in stressors, including discrimination and minority stress. However, little is known about differences in associations between avoidant coping and alcohol use among Black Americans and White Americans. Another study found that Black Americans who use avoidant coping are more likely to have alcohol-related consequences compared to White Americans (Cooper et al., 1992), consistent with overall findings about Black Americans having a higher likelihood of having alcohol-related consequences (Zapolski et al., 2014). Thus, when considering the association between PTSD and alcohol use, it is imperative to consider not only gender and racial differences to help explicate potential differences, but also to consider the use of avoidant coping to further understand the mechanisms underlying the association between PTSD and alcohol use.

Depressive symptoms are also an important factor to consider when examining the association between trauma, PTSD, and subsequent alcohol use. Sub-clinical levels of depressive symptoms on top of PTSD symptoms are a particular PTSD phenotype compared to individuals who have PTSD without sub-clinical depressive symptoms. Using a national sample, a positive association between depressive symptoms and alcohol use was found in the overall sample. However, there was no significant association between depression and alcohol consumption among African Americans (Keyes et al., 2011). It is unclear if subclinical depressive symptoms in combination with PTSD are differentially associated with alcohol use compared to individuals with PTSD and nonclinical depressive symptoms. It may be that individuals with both PTSD and depressive symptoms are more likely to use alcohol to cope with these mental health symptoms. Given that there are differential associations between depressive symptoms and alcohol use based on gender and race—and that these can inform critical prevention and intervention efforts—it is imperative to include the examination of depressive symptoms and individual differences when examining PTSD and alcohol use.

## Current study

The current study examined factors associated with alcohol use among emerging adults who had: no trauma exposure; trauma exposure and no PTSD; and trauma exposure with PTSD. We were particularly interested in testing unique factors associated with alcohol use among each of these populations to better understand the needs of emerging adults at risk for developing an alcohol use disorder. Risk factors examined in the current study focused on parental alcohol problems, depressive symptoms, and avoidant coping. Age and race were also included as covariates. Moreover, due to gender differences in alcohol use, we examined the independent effect of gender on alcohol use as well as the interaction between gender and avoidant coping in predicting alcohol use. We hypothesized that: (1) men would consume greater amounts of alcohol than women; (2) participants in the PTSD group would report higher levels of depressive symptoms and avoidant coping; (3) depressive symptoms and avoidant coping would be positivity associated with alcohol use; and (4) gender would interact with avoidant coping to predict greater alcohol use among men. Moreover, we predicted that the first, third, and fourth hypothesized effects listed above would be strongest among those in the PTSD group and weakest among those in the control group. Understanding differential risk factors for alcohol use is particularly needed among individuals who have been trauma-exposed (with or without PTSD), because alcohol use increases their likelihood of revictimization (Littleton & Ullman, 2013), may increase anxiety among people with PTSD, and can interfere with recovery (Straus et al., 2018).

## Method

### Participants

Participants of the current study were 238 young adults (59.2% women) between the ages of 21 and 30 years (*M* = 24.75, *SD* = 2.61) recruited through community advertisements. Participants were predominantly white (90.3% White; 9.7% Black). Participants were recruited to fill one of three study groups: non-trauma exposed (Control), trauma exposed without PTSD (Trauma Exposed), or trauma exposed with PTSD (PTSD). During an initial screening followed by a detailed baseline assessment, group assignment was determined using well-validated self-report measures, as well as a structured clinical interview with a trained interviewer (i.e., Life Events Checklist; Gray et al., 2004), Mini International Neuropsychiatric Interview (MINI; Sheehan et al., 1998), and the PTSD Checklist for *DSM-IV* (PCL; Weathers et al., 1993). Participants were eligible for the PTSD group if they had experienced at least one interpersonal traumatic event (i.e., trauma involving another person, including sexual assault/abuse; physical assault/abuse; witnessing violence) and met *DSM-IV* criteria for PTSD per the MINI and PCL. Participants were eligible for the Trauma Exposed (TE) group if they reported a history of at least one interpersonal traumatic event, did not meet criteria for PTSD during the structured clinical interview, and had a PCL score less than or equal to 25 (range: 17–85), which denotes the respondent was “asymptomatic” (Weathers et al., 2001). Participants were eligible for the Control group if they had no history of traumatic events (regardless of type of trauma) and did not meet criteria for PTSD. Participants who had a current diagnosis for an Axis I psychiatric disorder other than PTSD, including major depressive disorder or alcohol use disorder, or were currently taking antidepressants or anxiolytics, were excluded from participation in the study. Further details regarding the study participants and measures have been reported elsewhere (Danielson et al., 2021). Descriptive statistics for the three groups are reported in Table 1.

### Measures

#### Parent alcohol problems

Adults reported whether they believed that their mother or father had an alcohol use problem (Dube et al., 2001; O’Malley et al., 1986). Individuals who reported that they believed their parent had a problem were given a score of “1” and those who did not were given a score of “0.”

#### Depressive symptoms

Participants’ total score on the Beck Depression Inventory (BDI-II; Beck et al., 1996) was used to measure symptoms of depression (e.g., “I am so sad or unhappy that I can’t stand it”). The BDI-II is a well-validated and widely used measure of depression (Beck et al., 1996). Answer choices ranged from 0 to 4. Cronbach’s alpha for the BDI-II was 0.92.

#### Avoidant coping

Avoidant coping was assessed using mean scores of items on the behavioral disengagement, mental disengagement, denial, and substance use coping subscales of the COPE Inventory (Carver et al., 1989). Items were scored on a four-point Likert scale, with choices ranging from 1-“I usually don’t do this at all” to 4-“I usually do this a lot.” Psycho-metric properties of the COPE Inventory have been established through prior factor analytic work (Litman, 2006), indicating that these four subscales “hang together” to form this avoidant coping style. This subscale has been positively associated with other measures of avoidance-motivation (Litman, 2006). Cronbach’s alpha for the avoidant coping measure was 0.86.

#### Alcohol consumption

Alcohol consumption was assessed using the Alcohol Timeline Followback (TLFB; Sobell & Sobell, 1992), which is a valid, well-established, and most widely used measure of all forms of substance use, including drinking behavior. Participants were interviewed about their alcohol consumption over the past 30 days. The average number of drinks per day over the past 30 days was used for this investigation.

### Data analytic plan

In order to examine group differences, t-tests and chi-square analyses were conducted between the three study groups. Next, bivariate analyses were tested between groups (e.g., correlations). Finally, the a path model stacked on the three groups was run in Mplus 7.4 (Múthen & Múthen, 2015) using maximum likelihood parameter estimates with standard errors that are robust to non-normality and missing observations.

## Results

### Correlations

Table 2 provides correlation matrices for those in the PTSD, TE, and Control groups, respectively. Male participants reported more drinks per drinking day than female participants in all three groups. Avoidant coping was significantly associated with drinking in the TE (Trauma Exposed) and Control groups, but not in the PTSD group. Among people in the PTSD group, female gender was significantly associated with having a parent with an alcohol problem and greater levels of avoidant coping. Across all three groups, depressive symptoms were significantly associated with avoidant coping.

### Group path model

Age, gender, parent alcohol problem, race, depressive symptoms, and avoidant coping were entered as predictors, as well as a gender × avoidant coping interaction term. Because we were interested in examining differences between the three groups, we estimated a multiple-groups model. The model was first estimated with all paths constrained to be equal across the three groups. Nested model tests were conducted to examine if model fit improved by freeing the constraints on these paths, one at a time. Significant chi-square changes resulting from freeing the constraint on a given path indicates that the path is not equal between the groups. This resulted in freeing the paths from avoidant coping and the gender × avoidant coping interaction term. The model with all paths constrained demonstrated adequate fit: *X*^2^(14, *N* = 230) = 16.44, *p* =.287; RMSEA = 0.05 (90% *CI*[0.00, 0.13]); CFI = 0.95; SRMR = 0.083. After freeing the four aforementioned paths, the fit of the resulting model was significantly improved: *X*^2^(8, *N* = 230) = 5.21, *p* =.735; RMSEA = 0.00 (90% *CI*[0.00, 0.09]); CFI = 1.00; SRMR = 0.035.

Across all groups, gender was significantly associated with drinking, such that men had significantly higher alcohol use compared to women. Among participants in the Control group, the gender × avoidant coping interaction was significant such that avoidant coping was significantly associated with alcohol use, but only for men. Among participants in the TE and PTSD groups, avoidant coping had a significant effect on alcohol use over-and-above the effect of gender. Table 3 provides the standardized model results.

## Discussion

People often use alcohol or other substances to self-medicate in response to stress and traumatic events and the emotional distress that often follows them. However, over time this can exacerbate the aftereffects of traumatic event experiences and lead to worse psychological functioning (Straus et al., 2018). The present study investigated factors that may predict alcohol use in a sample of emerging adults who had varying levels of trauma exposure and impact, including those who reported no trauma exposure, trauma exposure without PTSD, and trauma exposure with PTSD. Consistent with previous research, young men in the current sample reported more drinks per day compared to young women across all three groups. Moreover, as hypothesized, participants in the PTSD group reported significantly higher depressive symptoms and avoidant coping compared to those in the TE and Control groups. Participants in the TE and PTSD groups were also significantly more likely to report a parent with an alcohol problem than participants in the Control group.

Interestingly, gender and avoidant coping also interacted to predict drinks per day, but this effect was only found in the Control group. Specifically, in the Control group, men were more likely to use alcohol, and that was attenuated at low levels of avoidant coping and strengthened at high levels of avoidant coping. Among both the TE and PTSD groups, avoidant coping had a significant effect on alcohol consumption over-and-above the effect of gender. This finding indicates that avoidant coping is an important predictor of alcohol use among people exposed to trauma, regardless of whether they have PTSD. Moreover, avoidant coping is also a significant predictor of alcohol use among males without trauma exposure. Emerging adults often engage in alcohol consumption in social situations (e.g., spending time with friends, going to bars, etc.). PTSD is often associated with avoiding potentially threatening situations, such as those just mentioned. Young people with PTSD who are avoiding these situations may have less opportunity for alcohol consumption than those who are not avoiding such places, events, and people. It is important to understand what drives behavior among the young adult population so as to help develop and better inform evidence-based intervention approaches, including personalized approaches.

While alcohol is frequently used to self-medicate and cope with feelings of distress associated with traumatic events, reducing alcohol consumption among trauma-exposed individuals is imperative for symptom improvement and prevention of further psychological, physiological, and social distress. Considering that alcohol misuse induces physiological stress (e.g., increased sympathetic activity, greater systemic inflammation) and can thereby lead to higher anxiety and depressive symptoms, the added effects of alcohol use over time could contribute to greater and prolonged PTSD symptoms and other mental health issues. Additionally, substance use problems can impair social functioning and result in less social support. Social support is imperative to psychological resilience following a stressor (Ozbay et al., 2007), and more support is associated with reduced severity of alcohol use disorder and greater retention among treatment-seeking patients (Dobkin et al., 2002).

An important finding of the current study is that avoidant coping had a significant effect on alcohol use among participants in the TE and PTSD groups. This is especially note-worthy given recent prospective research indicating that negative coping, a composite variable consisting of avoidant coping, is associated with greater alcohol use and greater PTSD symptoms one and two years later (Read et al., 2014). That is, if avoidant coping is not targeted through interventions, people with trauma exposure who engage this style of coping are likely to see increases in both PTSD symptoms and alcohol use as time progresses. Current evidence-based treatments for PTSD and trauma-exposure do not explicitly target avoidant coping. However, the current findings suggest that incorporating coping skills training into treatment may be effective in reducing alcohol use among people who have experienced interpersonal trauma.

To the best of our knowledge, the current study is the first to systematically examine factors associated with alcohol use among a sample comprised of young people with PTSD, people who were exposed to interpersonal trauma without PTSD, and those who had not experienced traumatic events. This design is a significant strength of the current study. Although many of the findings were consistent across groups, there were notable differences that could help inform evidence-based interventions. First, participants in the PTSD group reported significantly higher levels of depression and avoidant coping compared to those in the Control and TE exposed conditions. Given the strong associations between avoidant coping and negative outcomes, the higher levels of avoidant coping among people with PTSD is of high importance for intervention development. Moreover, among participants in the Control condition, avoidant coping was only significant in predicting alcohol use among young men. However, among those who had trauma exposure, including those with PTSD, avoidant coping was significant in predicting alcohol consumption over and above the effect of gender. That is, the design of the current study allowed for results that point to avoidant coping as a strong predictor of problematic alcohol use, especially among those with trauma exposure and PTSD.

### Limitations

Despite these findings, a few limitations should be noted. First, severity of PTSD symptoms may be an important factor contributing to alcohol use among participants in the PTSD group, which was not accounted for in the present study. Second, the sample was predominantly White, limiting statistical power for identifying differences by race. In addition, race-based stressors that disproportionately impact individuals from marginalized groups (i.e., discrimination and minority stress) were not assessed. Future research would substantially benefit from replicating these findings among a racially diverse sample and including discrimination and minority stress in the models, as well as other accounting for other forms of diversity (e.g., sexual minorities). Third, although we have observed the association between avoidant coping and alcohol use here, we did not assess their reasons for drinking alcohol. Future research that examines reasons for drinking qualitatively may shed light on predictors and allow for more precise intervention targets. Fourth, measurement of gender should be enhanced in future studies to help facilitate as easier disclosure of gender identities beyond woman or man. Lastly, participants were excluded if they had a current diagnosis for any psychiatric disorder other than PTSD, including major depressive disorder or alcohol use disorder, or were currently taking antidepressants or anxiolytics. Thus, results seen here are from a relatively healthy sample and may differ in populations with clinical depression, alcohol use disorder, or other related mental health disorder diagnoses.

### Clinical implications and summary

Results from the current study highlight the importance of avoidant coping as a risk factor for greater drinking behavior among young adults, particularly those who have experienced traumatic events. Given these associations among trauma exposure, PTSD, and avoidance, early prevention-focused interventions, as well as treatments, specifically targeting avoidant coping could have a significant impact in reducing co-occurring PTSD and alcohol use problems. Specifically, exposure-based interventions (e.g., Prolonged Exposure) serve as helpful ways to improve coping among young adults with trauma-related mental health symptoms as these interventions work to reduce the distress associated with traumatic memories through direct discussion of memories and unpairing the link between the memories and distress. Further, inteventions aimed toward building adequate coping skills (e.g., Dialectical Behavior Therapy [DBT]) could be especially effective in reducing co-occurring PTSD and alcohol use. DBT equips patients with skills, such as emotional regulation, distress tolerance, mindfulness, and interpersonal communication, which allows them to manage difficult emotions and cope with stress in healthier ways, reducing the urge to turn to alcohol. Combining this approach with an exposure-based, trauma-focused treatment can be particularly powerful, because it can address both the root cause of PTSD symptoms (the trauma itself) and the unhealthy coping mechanism (alcohol use) that can develop in response. With alarmingly high current rates of stress-related mental health and substance use problems in the United States, investments in lines of research that develop and evaluate (including best approaches to dissemination and implementation) of such interventions should be underscored as a public health priority (Danielson et al., 2023).

## Figures and Tables

**Table 1:** Descriptive statistics

Variable	Control (*N* = 85)			Trauma Exposed (*N* = 83)			PTSD (*N* = 70)		
	*M*	*SD*	Range	*M*	*SD*	Range	*M*	*SD*	Range
Age	24.78	2.59	21–30	24.86	2.60	21–30	24.59	2.69	21–30
Depressive Symptoms	3.09	6.89	0–48	4.37	3.79	0–15	14.49**	10.85	0–50
Avoidant Coping	1.49	0.32	1.00–2.88	1.56	0.34	1.06–3.07	1.99**	0.49	1.13–3.25
Drinks Per Day	1.51	1.42	0.16–8.32	1.76	1.67	0.20–8.24	1.69	1.54	0.19–7.93
Parent Alcohol Problem	21.2% endorsed; 78.8% denied			34.9% endorsed; 65.1% denied*			46.4% endorsed; 53.6% denied*		
Gender	57.7% Female; 42.3% Male			60.2% Female; 39.8% Male			61.4% Female; 38.6% Male		
Race	92.9% White; 7.1% Black			92.8% White; 7.2% Black			84.29% White; 15.7% Black		

* Denotes significant difference (*p* <.05) from control group;

** Denotes significant difference (*p* <.05) between the PTSD group and both control and trauma-exposed group

**Table 2:** Correlations among study variables

	1.	2.	3.	4.	5.	6.
PTSD Group						
1. Drinks Per Day						
2. Age	0.07					
3. Gender	0.35**	− 0.20				
4. Race	0.02	0.11	− 0.26*			
5. Parent Alcohol Problem	− 0.15	0.02	− 0.27*	0.03		
6. Depressive Symptoms	− 0.07	− 0.07	− 0.17	0.12	0.19	
7. Avoidant Coping	0.15	0.22	− 0.19*	0.18	0.10	0.50***
Trauma-Exposed Group						
1. Drinks Per Day						
2. Age	− 0.10					
3. Gender	0.35**	− 0.14				
4. Race	− 0.11	0.07	− 0.13			
5. Parent Alcohol Problem	0.14	0.04	0.02	− 0.01		
6. Depressive Symptoms	0.08	− 0.05	− 0.04	− 0.13	0.28*	
7. Avoidant Coping	0.26*	− 0.15	− 0.10	− 0.03	0.10	0.44***
Control Group						
1. Drinks Per Day						
2. Age	0.04					
3. Gender	0.32**	− 0.07				
4. Race	− 0.06	0.08	− 0.05			
5. Parent Alcohol Problem	0.12	0.11	0.08	0.19		
6. Depressive Symptoms	0.14	− 0.04	− 0.09	0.26*	0.11	
7. Avoidant Coping	0.31**	− 0.04	− 0.10	0.14	0.19	0.62***

Higher scores on all constructs correspond to higher values

0 = females, 1 = males; 0 = White, 1 = Black

Trauma exposed and control groups: 1 = parent alcohol problem, 0 = no parent alcohol problem

PTSD group *N* = 70; Trauma-exposed group *N* = 83; Control group *N* = 85

*Control Group*, Group with no trauma exposure, *Trauma-Exposed Group*, Group with trauma exposure and no PTSD, *PTSD Group*, Group with trauma exposure and PTSD

* *p* <.05,

** *p* <.01,

*** *p* <.001

**Table 3:** Model results predicting drinks per day (*N* = 230)

Predictor	Control		Trauma-exposed		PTSD Group	
	*B*	*SE*	*B*	*SE*	*B*	*SE*
Age^a^	0.04	0.06	0.03	0.05	0.04	0.05
Gender^a^	0.50***	0.09	0.42***	0.07	0.46***	0.08
Parent Alcohol Problem^a^	0.02	0.07	0.02	0.06	0.02	0.07
Race^a^	− 0.01	0.05	− 0.00	0.05	− 0.01	0.07
Depressive Symptoms^a^	− 0.04	0.08	− 0.02	0.03	− 0.06	0.11
Avoidant Coping	0.15	0.12	0.32**	0.10	0.34*	0.15
Gender × Avoidant Coping	0.38**	0.13	− 0.01	0.15	− 0.19	0.14

Gender (0 = Female, 1 = Male); Race (0 = White, 1 = Black); Parent Alcohol Problem (1 = parent alcohol problem, 0 = no parent alcohol problem). Predictors with superscript^a^ represent effects constrained across groups

*Control*, Group with no trauma exposure; *Trauma-exposed*, Group with trauma exposure and no PTSD; *PTSD Group*, Group with trauma exposure and PTSD; *B*, Standardized coefficient; *SE*, Standard error

* *p* <.05,

** *p* <.01,

*** *p* <.001

## Data Availability

The data used for these analyses are available upon request from the corresponding author.
